# Understanding Psychological Health in Adults with Irritable Bowel Syndrome: A Cross-Sectional Study Using the All of Us Research Program

**DOI:** 10.64898/2026.10.20.26365988

**Published:** 2026-10-21

**Authors:** Weizi Wu, Aolan Li, Jie Chen, Eunhea You, Peiyuan Liu, Leonel Rodriguez, Ming-Hui Chen, Xiaomei Cong

## Abstract

**Background:**

Psychological health is an important aspect of health and well-being among adults with irritable bowel syndrome, yet the interrelationships among individual vulnerabilities, social experiences, perceived stress, and psychological health outcomes remain poorly understood.

**Objective:**

To characterize psychological health and examine direct and indirect associations of childhood adversity and adult attention-deficit/hyperactivity disorder-related symptoms within an adapted Stress Process Model.

**Design:**

Cross-sectional secondary analysis.

**Setting:**

The United States All of Us Research Program.

**Participants:**

2,417 adults with electronic health record-documented irritable bowel syndrome and complete data on the primary analysis variables and covariates.

**Methods:**

Separate structural equation models examined childhood adversity and adult attention-deficit/hyperactivity disorder-related symptoms in relation to perceived stress, anxiety and depressive symptoms, and positive psychological well-being, with loneliness and perceived social support as psychosocial pathways. Models adjusted for sociodemographic and social-context factors.

**Results:**

The median age was 54.39 years; 80.7% were female and 84.4% were White. Moderate-to-high perceived stress was reported by 56.2%, at least moderate anxiety symptoms by 21.0%, and at least moderate depressive symptoms by 24.3%. Higher ACE-8 scores were associated with greater perceived stress, anxiety symptoms, and depressive symptoms and lower psychological well-being (β = 0.063, 0.089, 0.116, and -0.100, respectively); corresponding associations for higher ASRS-6 scores were β = 0.299, 0.355, 0.390, and -0.260. Loneliness-specific indirect associations were positive for perceived stress, anxiety, and depressive symptoms (ACE-8: β = 0.023-0.034; ASRS-6: β = 0.043-0.075) and negative for psychological well-being (ACE-8: β = -0.039; ASRS-6: β = -0.089; all p < .001). Social support-specific indirect associations were not significant in the ACE-8 model and were small and significant only for anxiety and depression in the ASRS-6 model (β = 0.003-0.004).

**Conclusions:**

Psychological health among adults with irritable bowel syndrome was associated with interconnected individual and social factors within an adapted Stress Process Model. Childhood adversity and adult attention-deficit/hyperactivity disorder-related symptoms showed direct and indirect associations with psychological health, with loneliness emerging as the most consistent social factor. These findings support prospective investigation of individual vulnerabilities and social experiences in psychological health. The cross-sectional design does not establish temporal ordering or causal mediation.

**What is already known:**

- Psychological distress is common in adults with irritable bowel syndrome.
- Loneliness and perceived social support describe distinct social experiences.

**What this paper adds:**

- Childhood adversity and adult attention deficit disorder with hyperactivity symptom burden were linked to psychological health.
- Loneliness was associated with distress and lower psychological well-being.

